# Genome sequence of influenza virus strain A/Ust-Kamenogorsk/972/2023 (H3N2) from Kazakhstan

**DOI:** 10.1128/mra.01063-25

**Published:** 2026-03-24

**Authors:** Nuray Ongarbayeva, Tatyana Glebova, Nailya Klivleyeva, Nurbol Saktaganov, Assem Baimukhametova, Mereke Kalkozhayeva, Galina Lukmanova, Kobey Karamendin

**Affiliations:** 1 Research and Production Center for Microbiology and Virology 374530, Almaty, Kazakhstan; Katholieke Universiteit Leuven, Leuven, Belgium

**Keywords:** human, H3N2, Kazakhstan, genome analysis, gene sequencing, strain

## Abstract

During the 2023 epidemic, circulation of the influenza virus subtype A/H3N2 was observed in East Kazakhstan. Here, we describe the complete genome sequence of the strain A/Ust-Kamenogorsk/972/2023 (H3N2) identified in a nasopharyngeal swab collected from a patient with signs of acute respiratory infection.

## ANNOUNCEMENT

*Alphainfluenzavirus influenzae* (Influenza A) viruses are enveloped, single-stranded RNA viruses with a segmented, negative-sense genome belonging to the family *Orthomyxoviridae*, genus *Alphainfluenzavirus* (1). Along with pandemic strains, seasonal and avian H3 strains are regularly identified in Kazakhstan (2–4), contributing to the evolution of this subtype. In 2023, the H3N2 strain dominated in all countries, particularly in Europe, Asia, and North America (5). This research describes one of the strains isolated during seasonal epidemics in Eastern Kazakhstan (49.57°N, 82.37°E, 26 December 2023). Monitoring for seasonal influenza virus strains and sequencing their genomes are essential for the regular updating of vaccines.

Viral RNA was extracted from a nasopharyngeal swab sample collected from a patient with signs of acute respiratory infection using the QIAamp Viral RNA Mini Kit (Qiagen, Germany) according to the manufacturer’s instructions. Preliminary viral cDNA synthesis was conducted, amplifying complete viral segments, including their ends, using conservative uni-12 and uni-13 primers in PCR (6). Library preparation was conducted using the MGIEasy PCR-Free FS DNA Library Prep Set (MGI, China) according to the manufacturer’s protocol. Sequencing was performed on an MGI DNBSEQ-G400 instrument using the MGI DNBSEQ-G400 Sequencing Set (MGI, China). Quality analysis of sequencing data was performed using FastQC (7). Default parameters were used for all software, unless otherwise specified. The sequence data obtained were trimmed at the 3′ and 5′ ends with an error probability limit of 0.05 using Geneious Prime v2025.2.2 software (Dotmatics, USA) (8). Assembly, mapping, and ORF prediction were conducted also using Geneious Prime (medium sensitivity, four iterations) against influenza A virus A/duck/Zhejiang/4613/2013 (H3N2) reference sequences downloaded from GenBank (accession numbers KF357795 to KF357818). The HA and NA sequences obtained were aligned (Clustal X algorithm), and phylogenetic trees were generated using the neighbor-joining method and the Tamura-Nei model with MEGA 12 (version 12.0.9) with 1,000 bootstrap replications to assign confidence levels for the branches automatically. The reference sequences were selected to represent the available clades of H3N2.

In total, 466,067 raw sequencing reads with a mean length of 130 nucleotides (nt) and guanine and cytosine (GC) content of 50.97% were obtained. The final obtained assembly contained 13,626 nucleotides with a mean coverage of 192.6-fold. The number of mapped reads constituted 21,238 reads (4.55%). The genome ends were determined when aligning to the reference genome. The deduced amino acid sequence of the hemagglutinin (HA) gene cleavage site revealed the presence of the PEKQTR*GIF motif that is typical for low-pathogenic influenza strains (9). BLASTn analyses revealed significant genetic similarity by all eight genes with the influenza viruses that circulated in Asia and North America (Table 1).

**TABLE 1:** Comparison of the nucleotide sequences of all genes of the Kazakhstan influenza A/H3N2 strain with the genetically most closely related strains in GenBank

Gene or segment	Gene size^*a*^ (nt)	GC content (%)	Most closely related strain	Identity with the most closely related strain at the nt level (%)	GenBank accession numbers for the most closely related strains
PB2	2341	43.0%	A/Human/New York City/PV98577/2023(H3N2)	99.8	PQ068011.1
PB1	2341	41.7%	A/Moscow/segment_2/2023(H3N2)	99.6	PQ632956.1
PA	2233	41.8%	A/USA/WA-UW-59573/2024(H3N2)	99.9	PP830129.1
HA	1762	41.9%	A/Human/New York City/PV98577/2023(H3N2)^*b*^	99.6	PQ068013.1
NP	1566	44.9%	A/Human/New York City/PV150138/2024(H3N2)	99.8	PQ068512.1
NA	1466	42.2%	A/Human/New York City/PV98577/2023(H3N2)^*c*^	99.7	PQ068015.1
M	1027	46.8%	A/Hong Kong/EPI0467/2024(H3N2)	99.8	PP535505.1
NEP, NS1	890	42.0%	A/Human/New York City/PV150138/2024(H3N2)	99.8	PQ068517.1

^*a*^ Complete or near-complete.

^*b*^ H3 clade 3C.2a.3a.1.

^*c*^ N2 clade B.4.

**Fig 1:**
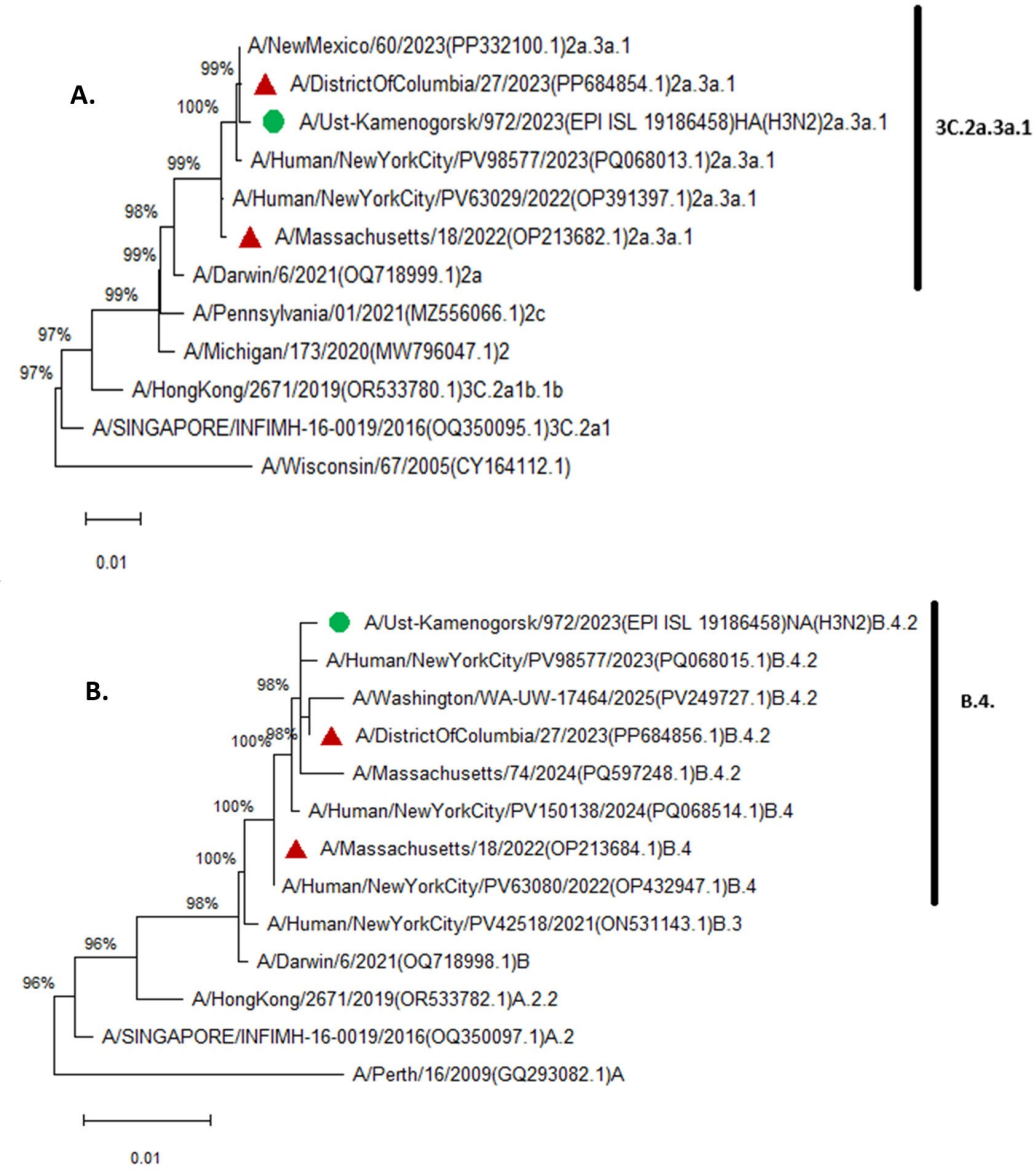
Phylogenetic tree of the hemagglutinin (**A**) and neuraminidase (**B**) genes of the A/Ust-Kamenologist/972/2023(H3N2) strain. The trees are drawn to scale, with branch lengths measured in the number of substitutions per site. The Kazakhstan strain of the H3N2 influenza virus is indicated by a green circle. Vaccine strains of the evolutionary lineages of the H3N2 influenza virus are indicated by red triangles.

Phylogenetic analysis has shown that the sequenced strain formed branch 3C.2a.3a.1 for the HA gene (Fig. 1A) and branch B.4 for the neuraminidase gene (Fig. 1B) . Genomic surveillance conducted worldwide revealed the emergence of A(H3N2) viruses belonging to clade 3C.2a.3a.1, which became nearly the exclusive H3 clade in 2023–2024 and 2024–2025 (10).

## Data Availability

The complete genome sequence of A/Ust-Kamenogorsk/972/2023 (H3N2) is publicly available at NCBI GenBank under accession numbers PX485628, PX485629, PX485630, PX485631, PX485632, PX485633, PX485634, and PX485635. The raw data generated for this genome have been deposited in the NCBI Sequence Read Archive (SRA) under accession number SRR35492372.
